# Risk Factors and Outcomes Associated With Delayed Villous Maturation in Placenta: A Systematic Review and Meta‐Analysis

**DOI:** 10.1111/1471-0528.70125

**Published:** 2026-01-12

**Authors:** Muhammad Pradhiki Mahindra, Muhammad Pradhika Mapindra, Hadi Waheed, Owen Vaughan, John Ciaran Hutchinson, Sara L. Hillman, Dimitrios Siassakos

**Affiliations:** ^1^ Maternal‐Fetal Medicine Department University College London Elizabeth Garrett Anderson Institute for Women's Health London UK; ^2^ Harapan Kita National Women and Children Centre Jakarta Indonesia; ^3^ Neonatology Department University College London Elizabeth Garrett Anderson Institute for Women's Health London UK; ^4^ Department of Pediatric and Perinatal Pathology Great Ormond Street Hospital London UK

**Keywords:** delayed villous maturation, distal villous immaturity, gestational diabetes, pregnancy outcomes, stillbirth

## Abstract

**Background:**

Delayed villous maturation (DVM) is a placental maturation disorder that mainly affects maternal‐to‐foetal oxygen transfer.

**Objectives:**

We conducted a systematic review, meta‐analysis, and sensitivity analysis exploring study heterogeneities (*I* ^*2*^) of risk factors and outcomes associated with histopathological findings of DVM.

**Search Strategy:**

Medline, EMBASE, Web of Science, and MIDIRS databases were searched from inception to December 2023.

**Selection Criteria:**

Peer‐reviewed, observational studies including cohort, case–control, and cross‐sectional studies reported the histopathological findings of DVM after placenta delivery. All eligible studies were included and assessed for their risk of bias using the Newcastle‐Ottawa scale (NOS) for cohort and case–control studies.

**Data Collection and Analysis:**

Two reviewers independently performed the systematic article screening, bias assessment, and data extraction. Senior authors resolved the disagreement between reviewers. The risk of bias was assessed by two reviewers using NOS criteria. The random‐effects model was used for meta‐analysis due to heterogeneity across studies. Sensitivity analyses were performed according to the NOS risk of bias assessment and the DVM definition per the Amsterdam criteria.

**Main Results:**

Fifty‐two eligible studies reporting DVM and linked risk factors and outcomes were included. The risk factors associated with DVM were gestational diabetes (GDM) (OR = 4.90; 95% CI = 2.98, 8.06; *I* ^2^ = 39%), pregestational diabetes (PGDM) (OR = 2.77; 95% CI = 1.56, 4.92; *I* ^2^ = 0%), and maternal obesity (OR = 1.88; 95% CI = 1.20, 2.96; *I* ^2^ = 0%). DVM was also associated with congenital foetal malformations (OR = 5.22; 95% CI =2.39, 11.39; *I* ^*2*^ = 40), stillbirth (OR = 4.89; 95% CI = 3.55, 6.72; *I* ^*2*^ = 0) and preterm birth (OR = 17.41; 95% CI = 10.14, 29.90; *I* ^2^ = 0). The association between DVM and stillbirth (OR = 12.06; 95% CI =3.40, 42.78; *I* ^2^ = 50; 2/5 studies) persisted in analyses limited to studies that used Amsterdam criteria exclusively for DVM.

**Conclusion:**

DVM is a placental abnormality associated with congenital foetal malformations and maternal dysmetabolism, including GDM, PGDM, and maternal obesity; and with adverse outcomes including stillbirth and preterm birth. In studies using Amsterdam criteria, placenta with DVM was associated with stillbirth and congenital malformations. Optimising metabolism could prevent harm to the baby.

## 1 Introduction

The formation of distal tertiary villi in the human placenta is controlled by trophoblast maturation and differentiation and is critical for uteroplacental blood flow, gas exchange, and nutrient transport. Pathological changes in the placental villous tree, known as villous maturational disorders, result in placental dysfunction by decreasing the formation of the vasculosyncytial membrane, the main site of maternal‐foetal transfer. Villous maldevelopment into the mature tertiary villi causes a disturbance in maternal‐foetal exchange, which leads to foetal asphyxia and distress [1, 2].

Distal villous immaturity is a maturational disorder of the placenta characterised by large distal villi with excessive stroma, an overabundance of trophoblastic layer, and scarcity of vasculosyncytial membrane [3]. Delayed villous maturation (DVM) is the most recent term used to define villous maturational disorders synonymous with DVI and characterised by villous vascular maturity not appropriately developing by gestational age, according to the Amsterdam Group Consensus Statement [4]. Overall, the umbrella term of villous immaturity related to DVM is broadly identified by crowding of intermediate mature villi with reduced presence of mature distal villi [3, 5, 6, 7, 8]. Additional histological characteristics associated with DVM are villous oedema, decreased vasculosyncytial membrane, reticular stroma, and centrally placed foetal capillaries [3, 4, 8, 9, 10, 11]. Immunohistochemistry using CD15+ staining has been reported as a possible endothelial marker to identify villous immaturity [12, 13].

Predicting the clinical relevance of DVM is challenging, as there are currently no recognised biochemical or imaging markers that identify its presence in the placenta. DVM can only be diagnosed during placental examinations post‐birth. Glucose dysmetabolism is often present in pregnancies complicated by DVM, but not at levels sufficient to drive a formal diagnosis of gestational diabetes [14]. Because DVM cannot be diagnosed prospectively, an effective strategy to prevent adverse outcomes associated with DVM would be to identify and address risk factors.

This systematic review and meta‐analysis aimed to gather and summarise the prevalence and pooled odds ratio of risk factors and outcomes associated with DVM findings in the placenta as well as to investigate associations when DVM was defined by Amsterdam consensus criteria, as compared to variable definitions of DVM including pre‐Amsterdam by sensitivity analysis.

## 2 Methods

### 2.1 Protocol, Search Strategy, and Eligibility Criteria

This review was conducted under the protocol recommendations of Preferred Reporting Items for Systematic Reviews and Meta‐Analysis (PRISMA) and Meta‐analysis of observational studies in epidemiology (MOOSE) [15, 16]. From electronic databases: MEDLINE (1946 to December 2023), EMBASE (1974 to December 2023), Web of Science (inception to December 2023), and Maternity & Infant Care Database (MIDIRS) (1971 to December 2023), the literature searching was performed using the combinations of the various keywords relevant to the proposed terms for the distal villous immaturity: ‘delayed villous maturation’, ‘villous maturation defect’, ‘variable villous maturation’, and ‘villous dysmaturity’ (Table S1). Various terms were used to reflect the different vocabularies and rules across different databases. This review has been registered with the PROSPERO database (registration number CRD42024490995).

Observational studies reporting singleton pregnancy with documented proportions of lesions relevant to the definition of placenta with distal villous immaturity [4, 5, 6, 7, 8, 9, 10, 11, 12, 17] were assessed for the eligibility criteria. In this review, we excluded conference abstracts and proceedings with no explicit peer review. Published studies not in English were discussed among the study authors, and consensus was reached on bias assessment and study inclusion. Studies with observational design, either case–control or cohort, were screened and assessed. As exposure in this review, we considered any reported risk factors or pregnancy outcomes, with histopathology reporting of DVM as the meta‐analysis outcome. Information on whether the involved pathologists were blinded to clinical information was also recorded. Study designs outside our scope (review articles, commentary, animal studies, in vitro studies, case reports, case series, clinical trials, randomised controlled trials, and birth cohorts) were excluded during the screening process. The study population included any singleton pregnancy with reported histopathology assessment for the placenta after delivery.

### 2.2 Record Screening

Screening of all records extracted from the electronic databases was independently reviewed by two review authors (MPI and MPA), based on the overview of the title and abstract. The reviewers are clinicians with experience and training in systematic review and meta‐analysis. The same two authors assessed the full texts of studies that met the eligibility criteria. The senior reviewer (DS) made the final decision on study inclusion when there was disagreement. From the included articles, the reference lists were hand‐searched by MPI and MPA using the snowballing method.

### 2.3 Quality Assessment

All potentially included studies were independently assessed for their quality by two review authors, based on the Newcastle Ottawa Scale (NOS) criteria and scoring system for cohort and case–control studies [18]. The method of quality assessment primarily emphasises 3 criteria: participant selection (4 stars), comparability (2 stars), and outcome/exposure assessment (3 stars). Studies were rated as low, moderate, or high risk of bias by awarding stars in the selection, comparability, and outcome domains, following the NOS guidelines. A “low risk” bias study required 3 or 4 stars in selection, 1 or 2 stars in comparability, and 2 or 3 stars in outcomes. A “moderate risk” study required 2 stars in selection, 1 or 2 stars in comparability, and 2 or 3 stars in outcomes. “High risk” studies were those that scored 0 or 1 star(s) in selection, or 0 stars in comparability, or 0 or 1 star(s) in outcomes [19].

### 2.4 Data Extraction and Evidence Synthesis

The study reviewers (MPI and MPA) independently extracted information from the included studies (author's name, publication year, study design, villous immaturity definition, study outcome definition, and the proportion of pregnancy events in the groups of placenta with and without DVM). From the included studies, we extracted the available data using a spreadsheet (Microsoft Excel Spreadsheet Software). Corresponding authors of potentially included studies were emailed directly for clarification of any unclear information needed for data extraction. If the authors did not respond to our request or provide the required information needed for our review and analysis, their studies were excluded.

Information was extracted regarding the proportion of pregnancy events reported with DVM and those without DVM, and the odds ratio for risk factors and outcomes associated with DVM was calculated. Maternal characteristics, including GDM and pregestational diabetes mellitus (PGDM—preexisting diabetes diagnosed before pregnancy), were also recorded. Foetal congenital malformations were also recorded. Stillbirth was defined as intrauterine foetal death ≥ 20 weeks until term, and preterm birth was defined as delivery of a baby < 37 weeks. We performed a meta‐analysis of risk factors and outcomes reported by at least 3 studies to avoid overestimating results from a single study.

### 2.5 Statistics, Data Analysis, and Meta‐Analysis

Due to methodological heterogeneity across studies, we used a random‐effects model in the meta‐analysis of proportions to estimate the pooled prevalence of adverse outcomes in pregnancies complicated by DVM. The effect measures of the extracted data from included studies were calculated using the pooled odds ratio (OR) and 95% confidence interval (CI). Statistical analysis and forest plot generation were conducted using Review Manager version 5.4. Interstudy variance was calculated based on the I‐squared statistic (*I* ^2^) value. An Egger test with *p* < 0.10, indicating a high risk of publication bias, was performed for each association. Egger test and funnel plots were generated using RStudio version 2024.12.1 + 563. Study heterogeneity and potential publication bias were explored by sensitivity analyses based on (1) NOS scoring system and (2) studies defining DVM per Amsterdam criteria compared to DVM using other definitions for each investigated study exposures, whereas *I* ^2^ of each association was also measured.

## 3 Results

**FIGURE 1:**
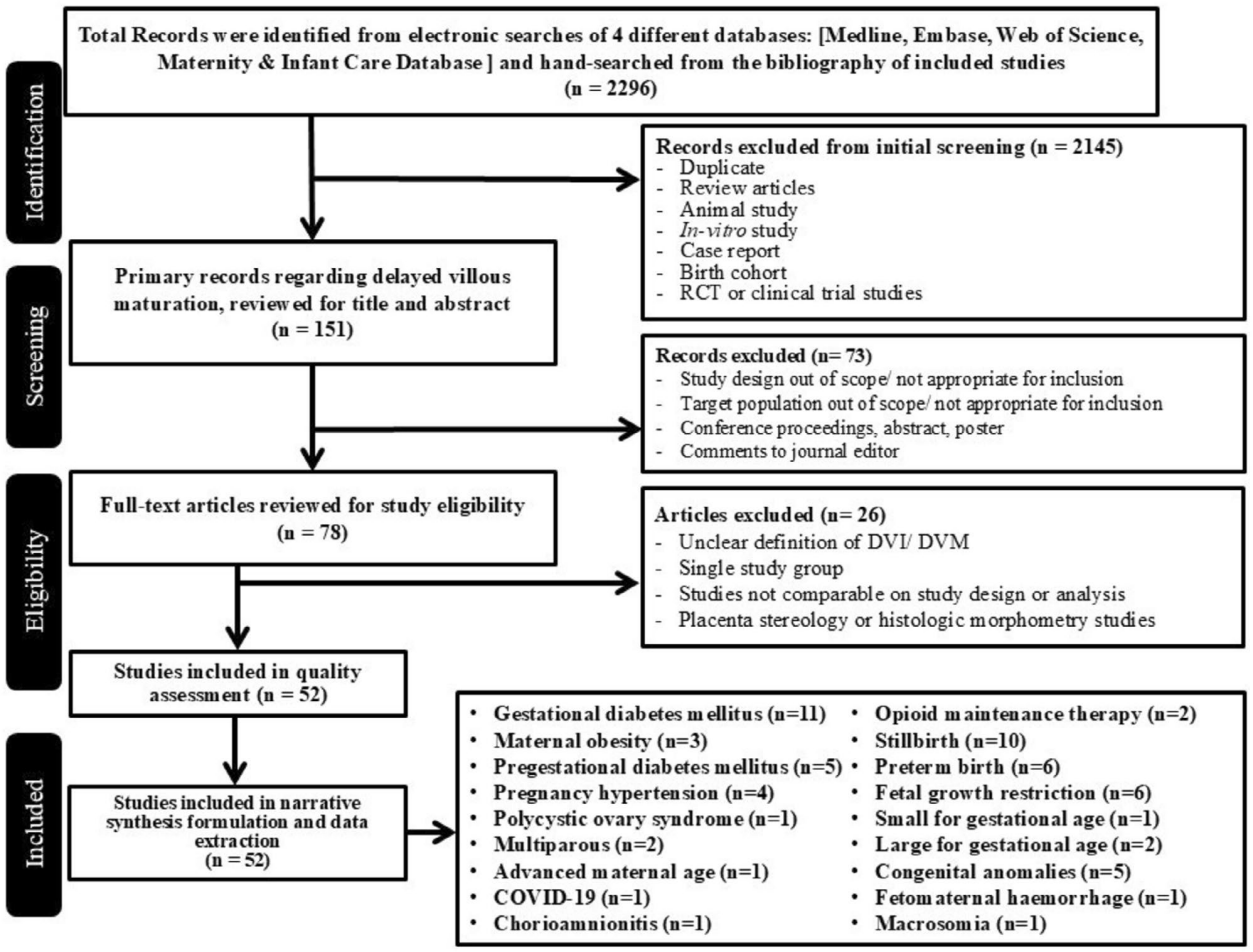
Flow chart of records screening and studies inclusion.

The overall study screening process can be seen in Figure 1. The search strategy yielded 2296 records from electronic searching, of which 2218 were excluded during the initial screening and abstract review due to duplicates, study designs not meeting inclusion criteria, study populations, and publication types. A total of 78 studies were assessed for quality, data extraction narrative formulation, and meta‐analysis synthesis. After excluding 26 studies of microscopic villous morphometry and single study groups following full‐text assessment, we finally obtained 52 included studies [2, 7, 14, 20, 21, 22, 23, 24, 25, 26, 27, 28, 29, 30, 31, 32, 33, 34, 35, 36, 37, 38, 39, 40, 41, 42, 43, 44, 45, 46, 47, 48, 49, 50, 51, 52, 53, 54, 55, 56, 57, 58, 59, 60, 61, 62, 63, 64, 65, 66, 67, 68, 69, 70], comprising 44 case–control studies and 8 cohort studies. The histopathological definitions of DVM used in the studies included in our review are presented in Table 1. There were no additional eligible studies from the snowballing method.

**TABLE 1:** Various definitions regarding the placenta with histopathology features of villous immaturity.

Villous immaturity reference	Definition used	Ref.
Amsterdam Placental Workshop Group Consensus Statement, 2016	The lesion of delayed villous maturation (DVM) is defined by a monotonous villous population (≥ 10 villi) with centrally placed capillaries and decreased vasculosyncytial membranes. The diagnosis is based on the presence of the lesion in at least 30% of 1 full‐thickness parenchymal slide.	[4]
Baergen, 2011	Villous immaturity was characterised by enlarged terminal villi with increased intervillous fluid, macrophages, and number of villous capillaries, which are located further from the villous syncytiotrophoblastic basement membrane than expected for gestational age	[9]
Benirschke and Kaufmann, 2000	Decreased formation of terminal villi and increased intermediate villi presence dependent on gestational age	[71]
Langston, 1997	Reticular villous stroma and central capillaries were observed in distal villi	[11]
De Laat, 2007	Reduced formation of vasculosyncytial membrane in terminal villi	[6]
Nagi, 2011	Villous maturity index score based on seven histopathology findings: (1) increased villous diameter (large distal villi), (2) thickened cellular layer of villous trophoblasts, (3) increased stromal cellularity, (4) prominent foetal capillaries, (5) rare or lack of vasculosyncytial membranes, (6) presence of interstitial fluid and (7) decreased distal/proximal villous ratio.	[8]
Redline, 2012	Immature villous is defined as: Typically: Increased immature intermediate/terminal villous ratio, effacement of the lobular architecture by increased distal, and immature villi appearance increased villous diameter, excessive cellular villous stroma, increased stromal extracellular matrix, centrally placed villous capillaries, hypercellular villous trophoblast with increased cytotrophoblast, and decreased vasculosyncytial membranes Commonly: Normal distal/proximal villous ratio, normal stem villi Occasionally: Increased syncytial knots, distal villous hypoplasia, and increased villous capillaries	[3]
Seidmann, 2014	According to immunophenotyping with CD15 staining, CD15‐positive vessels increase as villous maturity progresses. Negative staining of CD15 (CD15‐) denotes villous immaturity	[12]
Schweikhart, 1986	Placentas with predominant immature intermediate villi with rare presence of mature intermediate villi and distal villi	[7]
Vogel criteria of villous retardation, 1996	Villi with medium‐sized diameter, deficiently ramified into intermediate immature and mature villi. The stroma is cellular and reticular, with dense collagenous fibres, reduced capillaries, and deficient vasculosyncytial membranes.	[10]

### 3.1 Quality Assessment

The overall quality assessment, based on the Newcastle‐Ottawa scale for observational studies, is shown in Table 2. Using the Newcastle risk of bias criteria, 19 of 52 studies were considered high‐risk of bias because of a low score in the comparability domain for lack of multivariable adjustment.

**TABLE 2:** Quality assessment of the included studies.

Study ID	1	2	3	4	5	6	7	8	9	Selection	Comparability	Outcome	Interpretation of risk of bias
Ananthan 2019 [49]	*	*	*	0	0	*	*	*	*	3/4	1/2	3/3	Low risk
Arshad 2016 [20]	*	*	*	0	0	0	*	*	*	3/4	0/2	3/3	High risk
Bar 2017 [21]	*	*	*	0	*	*	*	*	*	3/4	1/2	3/3	Low risk
Beaudet 2007 [50]	*	*	*	0	0	*	*	*	*	3/4	0/2	3/3	Low risk
Bezemer 2020 [51]	*	*	*	0	*	*	*	*	?	3/4	1/2	2/3	Low risk
Bhattacharjee 2017 [22]	*	*	*	0	*	0	*	*	*	3/4	1/2	3/3	Low risk
Brouwers 2019 [23]^a^	*	*	*	*	*	*	?	*	*	4/4	2/2	2/3	Low risk
Bukowski 2017 [52]	*	*	*	0	*	0	*	*	*	3/4	1/2	3/3	Low risk
Burke 2023 [24]	*	*	*	0	0	*	*	*	*	3/4	1/2	3/3	Low risk
Castejon 2004 [25]	*	*	*	*	0	0	*	*	?	3/4	0/2	2/3	High risk
Corry 2016 [53]	*	*	*	0	*	*	*	*	*	4/4	1/2	3/3	Low risk
Danko 2023 [54]	*	*	*	0	0	0	*	*	0	3/4	0/2	2/3	High risk
Dasgupta 2022 [26]^a^	*	*	*	*	0	0	?	*	*	3/4	0/2	2/3	High risk
Daskalakis 2008 [27]	*	*	*	0	*	0	*	*	*	3/4	1/2	3/3	Low risk
De Noronha [55]^a^	*	*	*	*	0	0	*	*	*	4/4	0/2	3/3	High risk
Di Martino 2022 [28]	*	*	*	0	*	0	*	*	*	3/4	1/2	3/3	Low risk
Durhan 2017 [56]	*	*	*	0	0	*	*	*	*	3/4	1/2	3/3	Low risk
Evers 2003 [29]	*	*	*	0	*	0	*	*	*	3/4	1/2	3/3	Low risk
Freitag 1998 [57]	*	*	*	0	?	?	*	*	?	3/4	0/2	2/3	High risk
Giacometti 2023 [30]	*	*	*	0	*	*	*	*	*	3/4	2/2	3/3	Low risk
Higgins 2011 [31]	*	*	*	0	*	*	*	*	*	3/4	2/2	3/3	Low risk
Higgins 2012 [32]	*	*	*	0	0	0	*	*	*	3/4	0/2	3/3	High risk
Jaiman 2020 [72]	*	*	*	0	0	0	*	*	*	3/4	0/2	3/3	High risk
Jaiman 2021 [59]	*	*	*	0	*	0	*	*	*	3/4	1/2	3/3	Low risk
Jaiman 2022 [58]	*	*	*	0	*	?	*	*	*	3/4	1/2	3/3	Low risk
Kadivar 2020 [33]^a^	*	*	*	*	0	0	?	*	*	4/4	0/2	2/3	High risk
Kos 2005 [34]	*	*	*	0	0	0	?	*	*	3/4	0/2	2/3	High risk
Koster 2015 [35]	*	*	*	0	*	*	*	*	*	3/4	2/2	3/3	Low risk
Leavey 2017 [36]	*	*	*	*	*	0	*	*	*	4/4	1/2	3/3	Low risk
Lewis 2017 [61]	*	*	*	0	*	0	*	*	*	3/4	1/2	3/3	Low risk
Leon 2022 [60]^a^	*	*	*	*	*	0	?	*	0	4/4	1/2	1/3	High risk
Loardi 2016 [38]	*	*	*	*	*	*	*	*	*	4/4	2/2	3/3	Low risk
Madazli 2008 [39]	*	*	*	*	*	*	*	*	*	4/4	2/2	3/3	Low risk
Manocha 2019 [62]	*	*	*	0	?	?	*	*	*	3/4	0/2	3/3	High risk
Mehreen 2023 [63]	*	*	0	0	?	0	*	*	*	2/4	0.2	3/3	High risk
Moran 2014 [40]^a^	*	*	*	*	*	0	?	*	*	4/4	1/2	2/3	Low risk
O'Hare [64]^a^	*	*	*	*	0	0	?	*	*	4/4	0/2	2/3	High risk
Pacora 2019 [65]	*	*	*	0	*	0	*	*	*	3/4	1/2	3/3	Low risk
Pinar 2014 [66]	*	*	*	0	*	*	*	*	*	3/4	2/2	3/3	Low risk
Ruschowski 2020 [67]	*	*	*	*	0	*	*	*	*	4/4	1/2	3/3	Low risk
Schafer‐Graf 1997 [41]	*	*	*	*	0	*	*	*	?	4/4	1/2	2/3	Low risk
Schafer‐Graf 1998 [42]^a^	*	*	*	*	*	?	*	*	*	4/4	1/2	3/3	Low risk
Schweikhart 1986 [7]	*	*	*	0	0	?	*	*	*	3/4	0/2	3/3	High risk
Seidmann 2017 [70]	*	*	*	*	0	*	*	*	*	4/4	1/2	3/3	Low risk
Serra 2017 [43]	*	*	*	0	*	*	*	*	*	3/4	2/2	3/3	Low risk
Shanes 2020 [44]	*	*	*	0	0	0	*	*	*	3/4	0/2	3/3	High risk
Siassakos 2022 [14]	*	*	*	0	0	0	*	*	*	3/4	0/2	3/3	High risk
Stallmach 2001 [68]	*	*	*	*	0	?	*	*	0	4/4	0/2	2/3	High risk
Staszewski 2021 [45]	*	*	*	?	*	*	*	*	*	3/4	2/2	3/3	Low risk
Tasca 2021 [46]	*	*	*	0	*	*	*	*	*	3/4	2/2	3/3	Low risk
Torous 2020 [47]	*	*	*	0	?	*	*	*	*	3/4	1/2	3/3	Low risk
Treacy 2013 [69]	*	*	*	0	?	?	*	*	*	3/4	0/2	3.3	High risk

*Note:* The Newcastle‐Ottawa Scale for case–control studies: (1) Is the case definition adequate? (2) Representativeness of the cases. (3) Selection of controls. (4) Definition of controls. (5) Study controls for the most important factor. (6) Study controls for additional factors. (7) Ascertainment of exposure. (8) Same method of ascertainment for cases and controls. (9) Non‐response rate. * = star given; 0 = no star given; ? = unclear.

^a^ The Newcastle‐Ottawa Scale for cohort studies: (1) Representativeness of the exposed cohort. (2) Selection of the non‐exposed cohort. (3) Ascertainment of exposure. (4) Demonstration that outcome of interest was not present at start of study. (5) Study controls for the most important factor. (6) Study controls for additional factors. (7) Assessment of outcome. (8) Was follow‐up long enough for outcomes to occur. (9) Adequacy of follow‐up of cohorts.

### 3.2 Characteristics of the Included Studies

The characteristics of the included studies are shown in Table 3. The risk factors reported in association with increased incidence of histopathological findings of DVM were GDM, PGDM, maternal obesity, polycystic ovary syndrome, parity, hypertension, and also congenital malformations (Table 4). Congenital malformations consisted of congenital heart defects, gastroschisis, and postnatal diagnosis of Down syndrome following karyotype abnormalities. None of the studies reporting the association between congenital malformations and DVM documented cases with termination of pregnancy. The outcomes being reported in association with histopathological findings of DVM were stillbirth, spontaneous preterm birth, SGA (small for gestational age) babies, and LGA (large for gestational age) (Table 4).

**TABLE 3:** Characteristics of the included studies.

Study ID	Design	Case definition of DVM	Control	Blinded Yes/No	Risk factors	Outcomes	DVM vs non‐DVM placentas	OR (95% CI)
Ananthan 2019 [49]	Case–control	Baergen 2011 [9], Benirschke and Kaufmann 2000 [71]	Absent DVM	No		Stillbirth ≥ 20 weeks	33/47 vs. 52/123	3.22 (1.57–6.61)
Arshad 2016 [20]	Case–control	Benirschke and Kaufmann, 2000 [71]	Absent DVM	No	GDM		15/19 vs. 47/68	1.68 (0.50–5.66)
Bar 2017 [21]	Case–control	Society for Paediatric Pathology [3, 73]	Absent DVM	Yes	Obesity		46/70 vs. 123/262	2.17 (1.25–3.75)
Beaudet 2007 [50]	Case–control	Langston 1997 [11]	Absent DVM	Yes		SGA	30/96 vs. 216/1200	2.07 (1.31–3.27)
Bezemer 2020 [51]	Case–control	Amsterdam 2016 [4]	Absent DVM	Yes		SGA	0/0 vs. 250/292	Not estimable
						Stillbirth ≥ 20 weeks	6/6 vs. 58/100	9.44 (0.52–172.24)
Bhattacharjee 2017 [22]	Case–control	Benirschke and Kaufmann, 2000 [71]	Absent DVM	Yes	GDM		15/15 vs. 18/30	20.95 (1.15–383.02)
					PGDM		8/8 vs. 10/22	20.24 (1.04–393.62)
Brouwers 2019 [23]	Cohort	Amsterdam 2016 [4]	Absent DVM	Yes	Obesity		9/73 vs. 23/232	1.28 (0.56–2.90)
Bukowski 2017 [52]	Case–control	Redline 2012 [3]	Absent DVM	No		Stillbirth ≥ 20 weeks	32/54 vs. 282/1379	5.66 (3.24–9.89)
Burke 2023 [24]	Case–control	Amsterdam 2016 [4]	Absent DVM	Yes	Multiparous		6/9 vs. 116/386	4.66 (1.14–18.93)
Castejon 2004 [25]	Case–control	Benirschke and Kaufmann, 2000 [71]	Normal villous maturation	Yes	HDP		3/5 vs. 4/5	0.38 (0.02–6.35)
Corry 2016 [53]	Case–control	Amsterdam 2016 [4]	Absent DVM	No	Congenital malformations		21/29 vs. 31/75	3.73 (1.46–9.49)
Danko 2023 [54]	Case–control	Amsterdam 2016 [4]	Absent DVM	No		SGA	5/9 vs. 17/40	1.69 (0.39–7.26)
Dasgupta 2022 [26]	Cohort	Amsterdam 2016 [4]	Absent DVM	Yes	GDM		16/16 vs. 26/68	52.92 (3.05–919.54)
Daskalakis 2008 [27]	Case–control	Benirschke and Kaufmann, 2000 [71]	Absent DVM	Yes	GDM		32/51 vs. 8/29	4.42 (1.64–11.93)
De Noronha [55]	Cohort	Amsterdam 2016 [4]	Normal villous maturation	No	Congenital malformations		4/6 vs. 4/17	6.50 (0.85–49.69)
Di Martino 2022 [28]	Case–control	Amsterdam 2016 [4]	Absent DVM	Yes	HDP		17/23 vs. 19/29	1.49 (0.45–4.98)
Durhan 2017 [56]	Case–control	Amsterdam 2016 [4]	Absent DVM	Yes		SGA	14/16 vs. 11/39	17.82 (3.46–91.63)
Evers 2003 [29]	Case–control	Benirschke and Kaufmann, 2000 [71]	Absent DVM	Yes	PGDM		32/45 vs. 26/51	2.37 (1.01–5.52)
						LGA	23/45 vs. 17/51	2.09 (0.92–4.77)
Freitag 1998 [57]	Case–control	Vogel 1996 [10]	Absent DVM	Unclear		Preterm birth	28/40 vs. 36/104	4.41 (2.00–9.69)
Giacometti 2023 [30]	Case–control	Amsterdam 2016 [4]	Absent DVM	No	GDM		24/36 vs. 4/40	18 (5.19–62.44)
Higgins 2011 [31]	Case–control	Vogel 1996 [4, 10]	Absent DVM	Yes	GDM		15/175 vs. 6/175	2.64 (1.00–6.97)
					PGDM		14/175 vs. 5/175	2.96 (1.04–8.40)
					Multiparous		91/175 vs. 74/175	1.48 (0.97–2.25)
						Stillbirth ≥ 24 weeks	15/175 vs. 0/175	33.9 (2.01–571.13)
						Macrosomia	4/175 vs. 3/175	1.34 (0.30–6.08)
						Preterm birth	6/157 vs. 9/175	0.65 (0.23–1.88)
						SGA	17/175 vs. 36/175	0.42 (0.22–0.77)
Higgins 2012 [32]	Case–control	Vogel 1996 [4, 10]	Absent DVM	Yes	PGDM		21/32 vs. 53/119	2.38 (1.05–5.37)
Jaiman 2020 [72]	Case–control	Amsterdam 2016 [4]	Absent DVM	Yes		Stillbirth ≥ 20 weeks	31/35 vs. 112/513	27.75 (3.32–80.26)
Jaiman 2021 [59]	Case–control	Vogel 1996 [10]	Absent DVM	Yes		Preterm birth	62/68 vs. 271/707	16.62 (7.09–38.96)
Jaiman 2022 [58]	Case–control	Amsterdam 2016 [4]	Absent DVM	Yes		Preterm birth	30/43 vs. 65/571	17.96 (8.92–36.18)
Kadivar 2020 [33]	Cohort	Amsterdam 2016 [4]	Absent DVM	Yes	GDM		49/93 vs. 68/129	1 (0.59–1.71)
Kos 2005 [34]	Case–control	Benirschke and Kaufmann, 2000 [71]	Absent DVM	No	HDP		6/6 vs. 273/1683	67.04 (3.77–1193.57)
Koster 2015 [35]	Case–control	De Laat, 2007 [6]	Absent DVM	Yes	PCOS		30/78 vs. 43/204	2.34 (1.33–4.12)
Leavey 2017 [36]	Case–control	Amsterdam 2016 [4]	Normal villous maturation	Yes	HDP		4/17 vs. 15/50	0.72 (0.20–2.56)
						SGA	3/17 vs. 3/50	3.36 (0.61–18.52)
					Chorioamnionitis		5/17 vs. 7/50	2.56 (0.69–9.52)
Leon 2022 [60]	Cohort	Amsterdam 2016 [4]	Absent DVM	No	Congenital malformations		13/13 vs. 22/62	48.6 (2.76–856.58)
Lewis 2017 [61]	Case–control	Baergen 2011 [9]	Absent DVM	Yes	Fetomaternal haemorrhage		5/6 vs. 30/108	13 (1.5–115.9)
Loardi 2016 [38]	Case–control	Benirschke and Kaufmann, 2000 [71]	Normal villous maturation	Yes	Obesity		2/2 vs. 8/18	6.18 (0.26–146.78)
Madazli 2008 [39]	Case–control	Baergen 2011 [9]	Normal villous maturation	Yes	GDM		13/15 vs. 9/29	14.44 (2.68–77.80)
Manocha 2019 [62]	Case–control	Amsterdam 2016 [4]	Absent DVM	No		Preterm birth	9/11 vs. 76/89	0.8 (0.1–4.0)
Mehreen 2023 [63]	Case–control	Amsterdam 2016 [4] and CD15 by immunohistochemistry	Absent DVM	Yes		LGA	151/435 vs. 1087/8711	3.7 (3.0–4.6)
Moran 2014 [40]	Cohort	Amsterdam 2016 [4]	Absent DVM	Unclear	PGDM		9/12 vs. 37/57	1.62 (0.39–6.68)
O'Hare [64]	Cohort	Amsterdam 2016 [4]	Absent DVM	Yes	Congenital malformations		25/26 vs. 99/137	9.6 (1.3–73.3)
Pacora 2019 [65]	Case–control	Amsterdam 2016 [4]	Absent DVM	Yes		Stillbirth ≥ 20 weeks	12/15 vs. 46/103	5 (1.3–18.6)
Pinar 2014 [66]	Case–control	Baergen 2011 [9], Benirschke and Kaufmann 2000 [71](99)	Absent DVM	No		Stillbirth ≥ 20 weeks	53/75 vs. 465/1409	4.9 (2.9–8.1)
Ruschowski 2020 [67]	Case–control	Amsterdam 2016 [4]	Normal villous maturation	Yes	Congenital malformations		15/18 vs. 8/27	11.9 (2.7–52.7)
Schafer‐Graf 1997 [41]	Case–control	Vogel 1996 [10]	Normal villous maturation	Yes	GDM		67/82 vs. 61/111	3.66 (1.87, 7.18)
Schafer‐Graf 1998 [42]	Case–control	Vogel 1996 [10]	Normal villous maturation	Yes	GDM		75/94 vs. 66/124	3.47 (1.88–6.41)
Schweikhart 1986 [7]	Case–control	Schweikhart 1986 [7]	Absent DVM	Unclear		Preterm birth	36/81 vs. 74/596	5.6 (3.4–9.3)
						Stillbirth ≥ 29 weeks	6/31 vs. 9/70	1.6 (0.5–5.1)
Seidmann 2017 [70]	Case–control	Vogel 1996 [10] and immunostaining with CD15+/ CD31+/ CD34+	Normal villous maturation	Yes		SGA	4/65 vs. 2/37	1.1 (0.2–6.6)
Serra 2017 [43]	Case–control	Amsterdam 2016 [4]	Absent DVM	No	Opioid maintenance therapy		36/92 vs. 318/1240	1.86 (1.20–2.89)
Shanes 2020 [44]	Case–control	Amsterdam 2016 [4]	Absent DVM	No	Maternal infection		4/621 vs. 11/1183	0.69 (0.22–2.18)
Siassakos 2022 [14]	Case–control	Amsterdam 2016 [4]	Absent DVM	No	GDM		7/8 vs. 3/5	4.67 (0.30–73.38)
Stallmach 2001 [68]	Case–control	Vogel 1996 [10]	Normal villous maturation	No		Stillbirth 32–42 weeks	23/993 vs. 575/16422	0.7 (0.4–1.0)
Staszewski 2021 [45]	Case–control	Amsterdam 2016 [4]	Unclear	Unclear	Opioid maintenance therapy		37/41 vs. 66/102	5.05 (1.66–15.29)
Tasca 2021 [46]	Case–control	Amsterdam 2016 [4]	Absent DVM	No	COVID‐19		9/15 vs. 55/113	1.58 (0.53–4.74)
Torous 2020 [47]	Case–control	Amsterdam 2016 [4]	Absent DVM	No	Advanced maternal age		14/19 vs. 154/209	1 (0.34–2.91)
Treacy 2013 [69]	Case–control	Redline 2012 [3]	Absent DVM	No		Stillbirth ≥ 35 weeks	12/12 vs. 1/16	258.33 (9.66–6908.03)

Abbreviations: DVM, delayed villous maturation; GDM, gestational diabetes mellitus; HDP, hypertension during pregnancy; LGA, large for gestational age; OST, opioid substitute therapy; PCOS, polycystic ovary syndrome; PGDM, pregestational diabetes mellitus; SGA, small for gestational age; SGA, small for gestational age.

**TABLE 4:** Studies reporting increased incidence of DVM.

Risk factors and outcomes	Study author	Definitions of DVM	Blinded (Y/N)	Number of pathologist involved	Number of DVM in exposed group (%)	Number of DVM in non‐exposed group (%)	Ref
GDM	Bhattacharjee 2017	Villous immaturity was defined as when there was a decreased formation of terminal villi and a relatively increased presence of immature intermediate villi.	Yes	> 1	15/33	0/12	[22]
	Dasgupta 2022	Immature villi were recognised by their characteristic morphological features: enlarged terminal villi with excessive Stroma, hypercellular villous trophoblasts, paucity of vasculosyncytial membranes	Yes, except for the gestational age	> 1	16/42	0/42	[26]
	Daskalakis 2008	Villous immaturity was defined when there was decreased formation of terminal villi and increased presence of immature intermediate villi in relation to gestational age	Yes, except for the gestational age	1	32/40	19/40	[27]
	Giacometti 2023	Amsterdam placental workshop group consensus statement [4]	No	> 1	24/28	12/48	[30]
	Higgins 2011	Delayed villous maturation was defined as villi with decreased vasculosyncytial membranes, a continuous trophoblast covering, and increased stroma; more severe grades showed decreased tertiary villi and the presence of large bulbous villi	Yes	1	15/21	160/329	[31]
	Madazli 2008	Villous immaturity was defined when there was a relatively decreased formation of terminal villi and a relatively increased presence of mature and immature intermediate villi in relation to gestational age in each slide.	Yes	1	13/22	2/22	[39]
	Schafer‐Graf 1997	Vogel criteria for villus retardation [10]	Yes	1	67/128	15/65	[41]
	Schafer‐Graf 1998	Vogel criteria for villus retardation [10]	Yes	1	75/141	19/77	[42]
PGDM	Bhattacharjee 2017	Villous immaturity was defined as when there was a decreased formation of terminal villi and a relatively increased presence of immature intermediate villi.	Yes	> 1	8/18	0/12	[22]
	Evers 2003	Villous immaturity was defined as the relatively decreased formation of terminal villi and a relatively increased presence of immature and/or mature intermediate villi in relation to gestational age.	Yes	1	32/58	13/38	[29]
	Higgins 2011	Delayed villous maturation was defined as villi with decreased vasculosyncytial membranes, a continuous trophoblast covering, and increased stroma; more severe grades showed decreased tertiary villi and the presence of large bulbous villi	Yes	1	14/19	161/331	[31]
	Higgins 2012	Delayed villous maturation defined as a tertiary villus with decreased vasculosyncytial membranes, a continuous cuboidal trophoblast covering and increased villous stroma compared to normal	Yes	1	21/74	11/77	[32]
Obesity	Bar 2017	Society for Paediatric Pathology [73]	Yes	1	46/169	24/163	[21]
PCOS	Koster 2015	Villous immaturity was defined as a delay in the formation of syncytiocapillary membranes in terminal villi [6]	Yes	1	30/73	48/209	[35]
Multiparous	Burke 2023	Amsterdam placental workshop group consensus statement [4]	Yes	1	6/122	3/273	[24]
HDP	Kos 2005	Benirschkeand Kaufmann, 2000 [71]	No	1	6/279	0/1410	[34]
Opioid maintenance therapy	Serra 2017	Amsterdam placental workshop group consensus statement [4]	No	1	36/56	354/978	[43]
	Staszewski 2021	Amsterdam placental workshop group consensus statement [4]	No	> 1	37/103	4/40	[45]
Stillbirth	Ananthan 2019	Baergen 2011 [9], Benirschke and Kaufmann 2000 [71]	No	> 1	33/85	14/85	[49]
	Bukowski 2017	Distal villous immaturity is characterised by enlarged distal villi with excessive stroma, hypercellular villous trophoblast, paucity of Vasculosyncytial membranes, and a decreased fetoplacental weight ratio [3].	No	> 1	32/314	22/1119	[52]
	Higgins 2011	Delayed villous maturation was defined as villi with decreased vasculosyncytial membranes, a continuous trophoblast covering, and increased stroma; more severe grades showed decreased tertiary villi and the presence of large bulbous villi	Yes	1	15/15	160/335	[31]
	Jaiman 2020	DVM is defined by the presence of a monotonous villous population (at least 10 such villi) with centrally placed capillaries and decreased vasculosyncytial membranes, Recapitulating the histology of early pregnancy and involving 30% of one full‐thickness parenchymal slide [4]	Yes	1	31/143	4/405	[72]
	Pacora 2019	Amsterdam placental workshop group consensus statement [4]	Yes	1	12/58	3/60	[65]
	Pinar 2014	Baergen 2011 [9], Benirschke and Kaufmann 2000 [71]	No	> 1	53/518	22/966	[66]
	Treacy 2013	Redline 2012 [3]	No	> 1	12/13	0/15	[69]
Spontaneous preterm birth	Freitag 1998	Vogel criteria for villus retardation [10]	Unclear	Unclear	28/64	12/80	[57]
	Jaiman 2021	Amsterdam placental workshop group consensus statement [4]	Yes	> 1	62/333	6/442	[59]
	Jaiman 2022	The hallmarks of immature villi include [1] the presence of centrally placed capillaries, [2] a relatively large amount of stroma, and [3] fewer and less well‐formed vasculosyncytial membranes, in at least one‐third of a slide.	Yes	> 1	30/95	13/519	[58]
SGA	Durhan 2017	Amsterdam placental workshop group consensus statement [4]	Yes	1	14/25	2/30	[56]
	Beaudet 2007	Langston 1997 [11]	Yes	1	30/246	66/150	[50]
FMH	Lewis 2017	Villous immaturity was characterised by enlarged terminal villi with increased intravillous fluid, macrophages, and numbers of villous capillaries, which are located further from the villous syncytiotrophoblastic basement membrane than expected for gestational age [9]	Yes	1	5/35	1/79	[61]
LGA	Mehreen 2023	Amsterdam placental workshop group consensus statement [4] and immunohistochemistry findings of CD15	Yes	1	151/1238	284/7908	[63]
Down syndrome	Corry 2016	Amsterdam placental workshop group consensus statement [4]	No	> 1	21/52	8/52	[53]
CHD	Leon 2022	Amsterdam placental workshop group consensus statement [4]	No	> 1	13/35	0/40	[60]
CHD	O'Hare 2023	Amsterdam placental workshop group consensus statement [4]	Yes	> 1	25/124	1/39	[64]
Gastroschisis	Ruschowski 2020	Amsterdam placental workshop group consensus statement [4]	Yes	> 1	15/23	3/22	[67]

Abbreviations: CHD, congenital heart disease; DVM, delayed villous maturation; FMH, fetomaternal haemorrhage; GDM, gestational diabetes mellitus; LGA, large for gestational age; PCOS, polycystic ovary syndrome; PGDM, pregestational diabetes mellitus; SGA, small for gestational age; SGA, small for gestational age.

### 3.3 Meta‐Analysis

**FIGURE 2:**
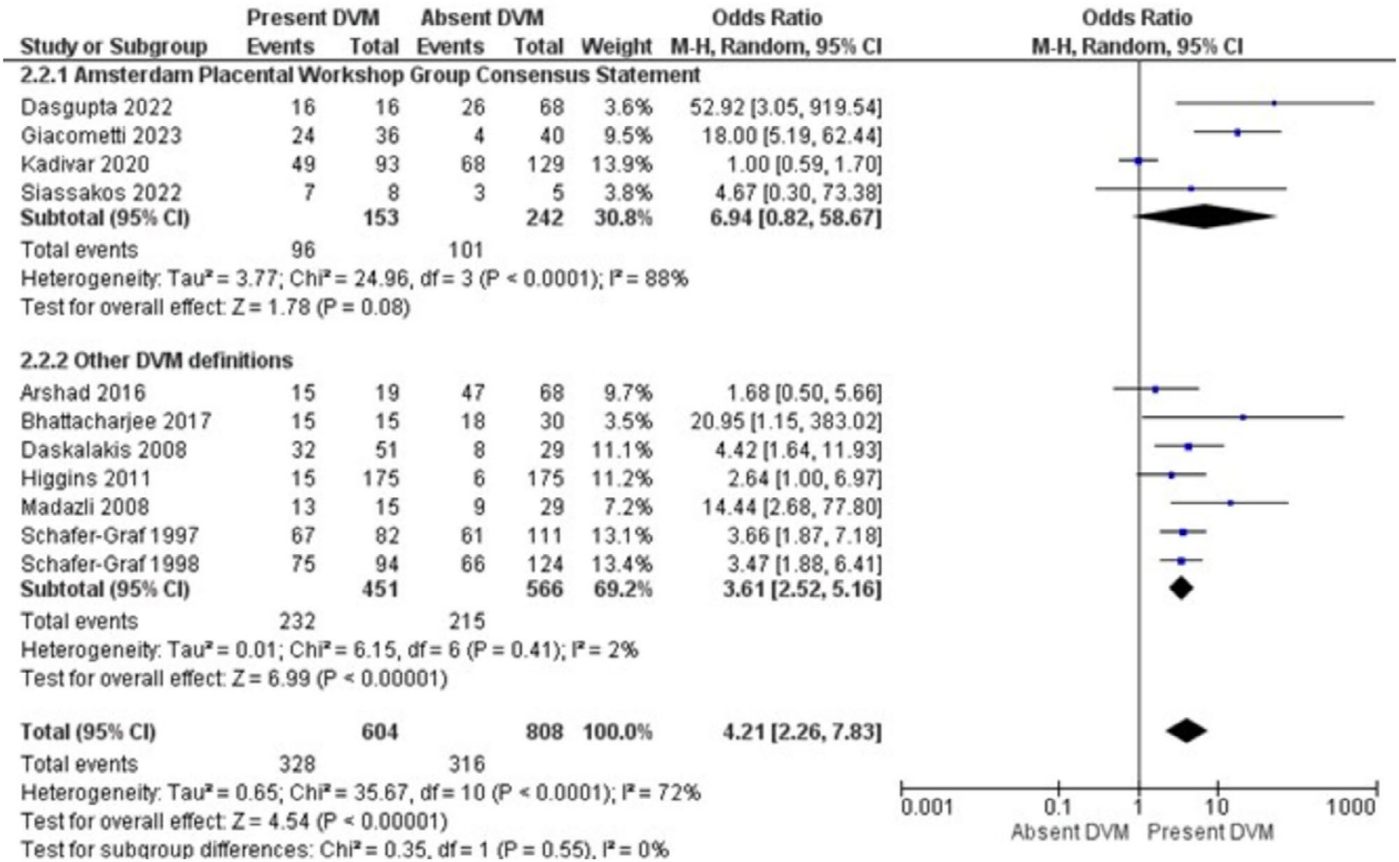
Forest Plot—Gestational Diabetes Mellitus and DVM.

**FIGURE 3:**
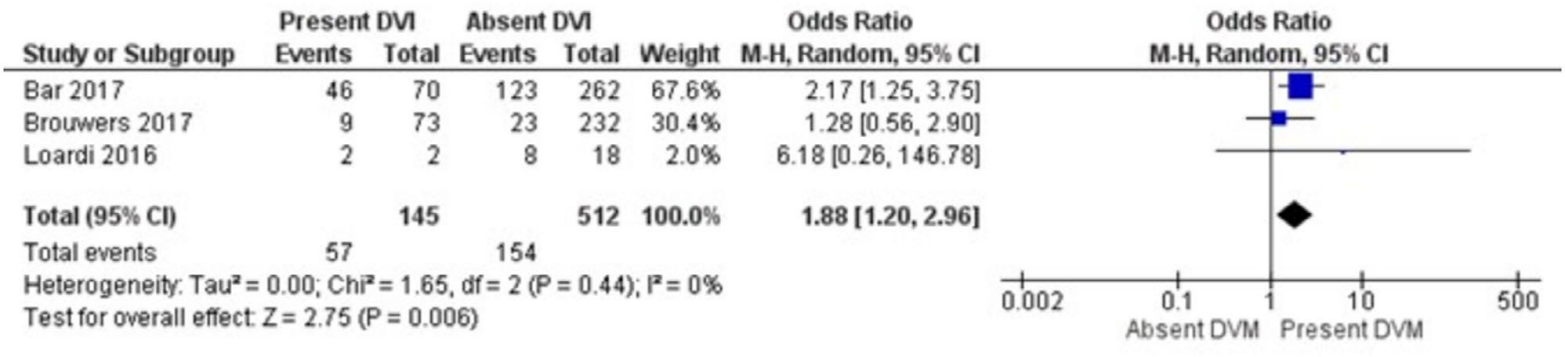
Forest Plot—Maternal Obesity and DVM.

**FIGURE 4:**
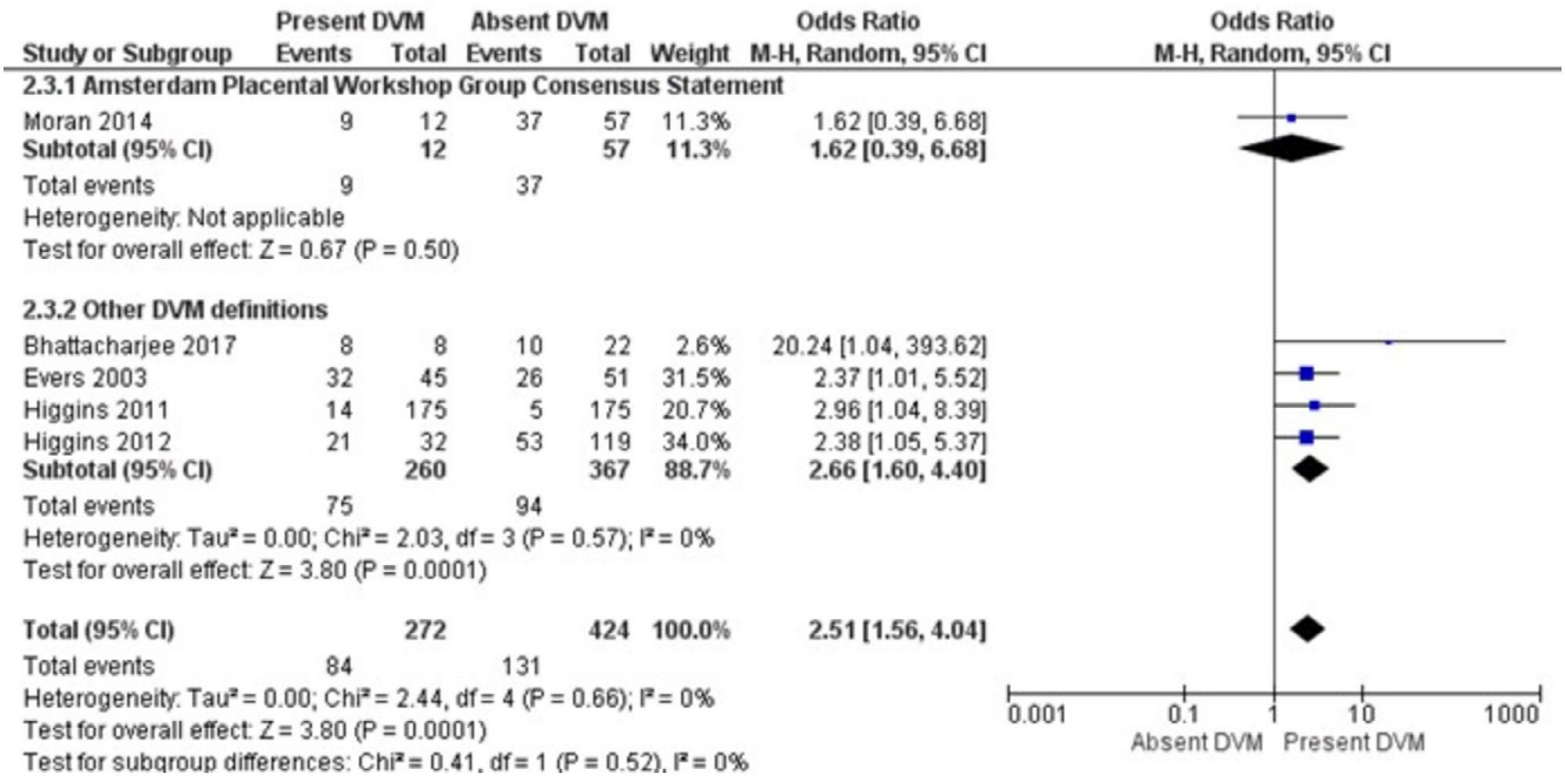
Forest Plot—Pregestational Diabetes Mellitus and DVM.

**FIGURE 5:**
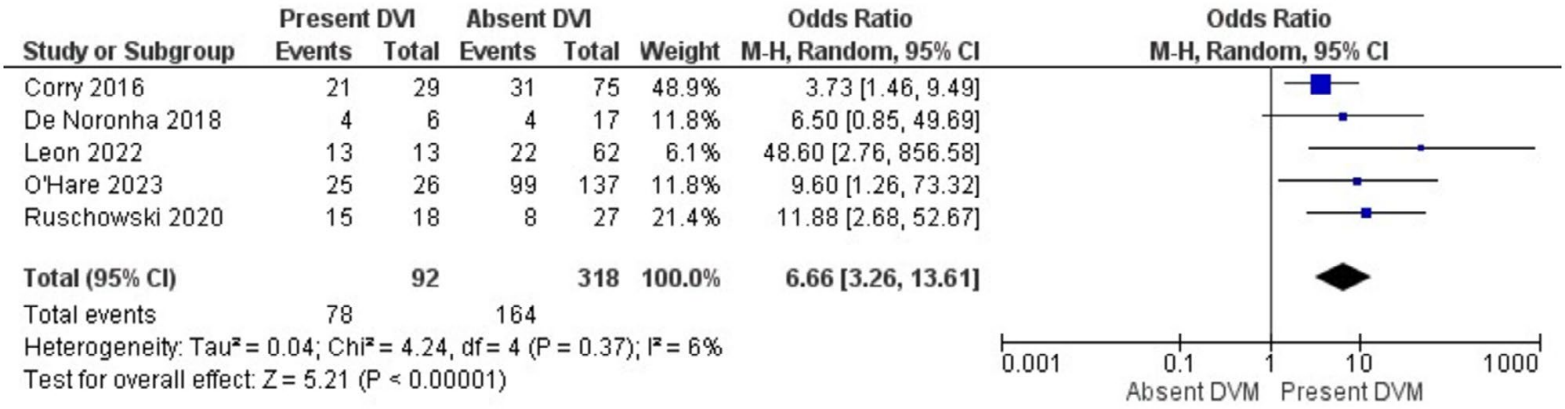
Forest Plot—Congenital Malformations and DVM.

**FIGURE 6:**
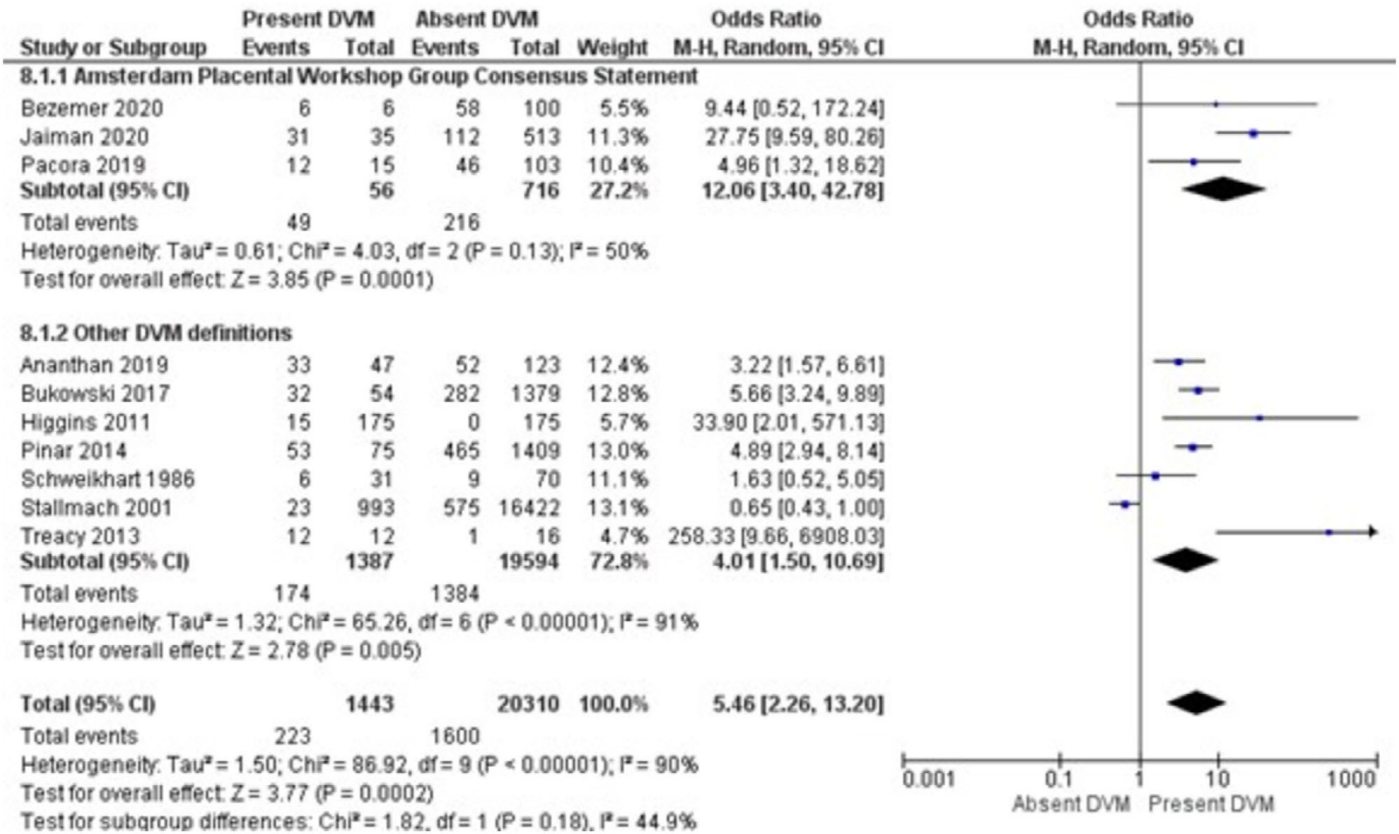
Forest Plot—Stillbirth and DVM.

**FIGURE 7:**
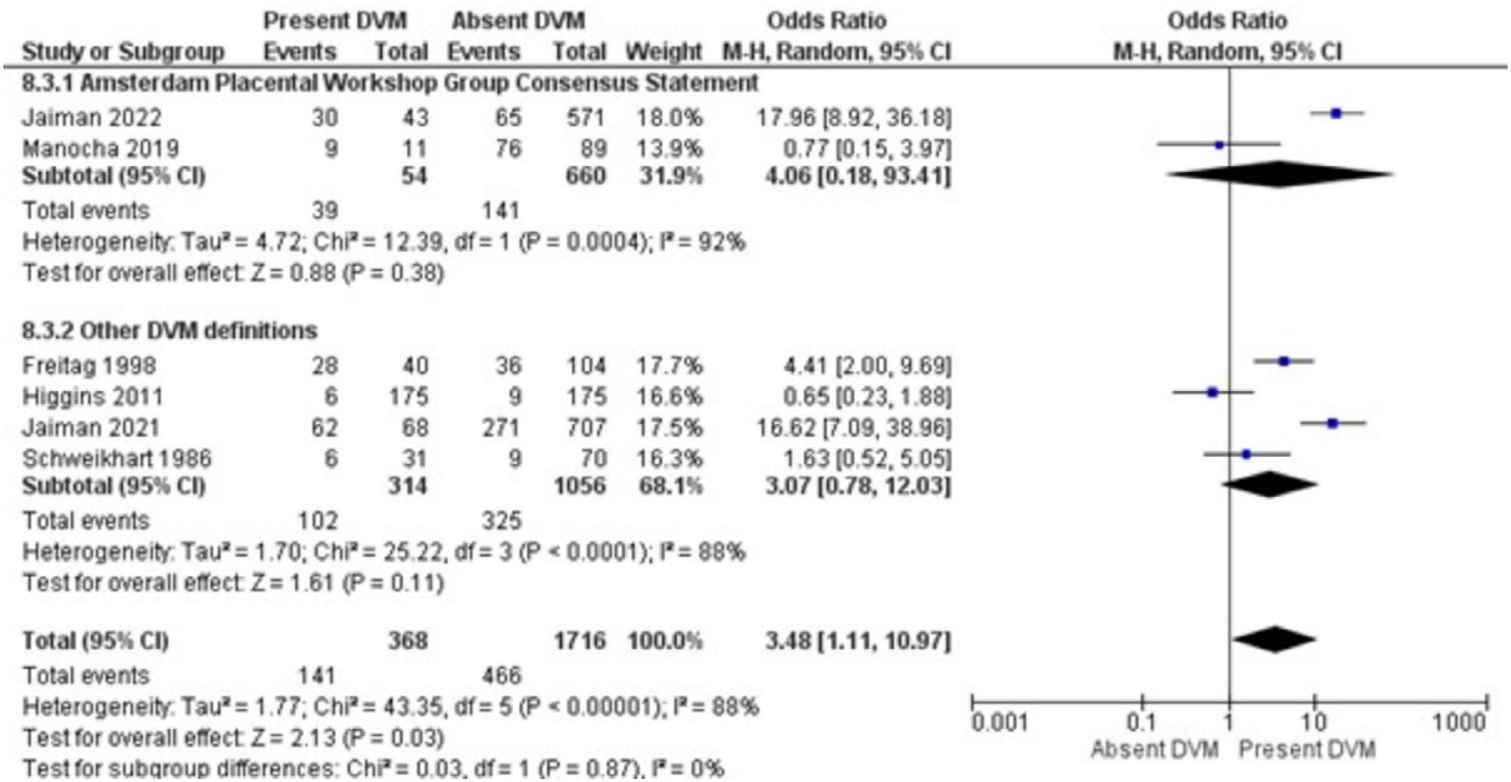
Forest Plot—Preterm Birth and DVM.

The results of the meta‐analysis and sensitivity analysis for each investigated risk factor and outcomes are displayed in Table 5. GDM (OR = 3.75; 95% CI = 1.93, 7.29; *I* ^2^ = 75%), PGDM (OR = 2.51; 95% CI = 1.56, 4.04; *I* ^2^ = 0%), maternal obesity (OR = 1.88; 95% CI = 1.20, 2.99; *I* ^2^ = 0%), and foetal congenital malformations (OR = 6.66, 95% CI =3.26, 13.61; *I* ^2^ = 6%) were the risk factors associated with increased pooled odds of DVM (Figures 2, 3, 4, 5). Stillbirth (OR = 5.46; 95% CI = 2.26, 13.20; *I* ^2^ = 90%) and preterm birth (OR = 3.48; 95% CI = 1.11, 10.97; *I* ^2^ *= 88%*) were the perinatal outcomes associated with DVM (Figures 6, 7).

**TABLE 5:** Meta‐analysis and sensitivity analyses.

Risk factors and outcomes	Pooled OR (95% CI), *I* ^2^	Publication bias by Egger's test	Sensitivity Analysis limited to studies with low risk of bias score—Pooled OR (95% CI), *I* ^2^	Sensitivity Analysis limited to studies using Amsterdam criteria—Pooled OR (95% CI), *I* ^2^
Gestational diabetes mellitus	3.75 (1.93, 7.29)^a^ *I* ^2^ = 75%	*z*‐score 2.63 *p*‐value < 0.01^b^	4.90 (2.98, 8.06)^a^ *I* ^2^ = 39%	6.94 (0.82, 58.67) *I* ^2^ = 88%
Obesity	1.88 (1.20, 2.96)^a^ *I* ^2^ = 0%	*z*‐score 0.49 *p*‐value 0.62	N/A	No studies
Pregestational diabetes	2.51 (1.56, 4.04)^a^ *I* ^2^ = 0%	*z*‐score 1.03 *p*‐value 0.30	2.77 (1.56, 4.92)^a^ *I* ^2^ = 0%	1.62 (0.39, 6.68) *I* ^2^ = N/A
Hypertensive disorders in pregnancy	1.80 (0.36, 9.07) *I* ^2^ = 68%	*z*‐score 0.73 *p*‐value 0.46	1.06 (0.44, 2.53) *I* ^2^ = 0%	1.66 (0.44, 2.53) *I* ^2^ = 0%
Stillbirth	5.46 (2.26, 13.20)^a^ *I* ^2^ = 90%	*z*‐score 2.42 *p*‐value 0.01^b^	4.89 (3.55, 6.72)^a^ *I* ^2^ = 0%	12.06 (3.40, 42.78)^a^ *I* ^2^ = 50%
SGA	1.69 (0.55, 5.21) *I* ^2^ = 80%	*z*‐score −0.06 *p*‐value 0.95	1.41 (0.30, 6.57) *I* ^2^ = 18%	1.84 (0.50, 6.76) *I* ^2^ = 84%
Preterm birth	3.48 (1.11, 10.97)^a^ *I* ^2^ = 88%	*z*‐score −1.83 *p*‐value 0.07^b^	17.41 (10.14, 29.90)^a^ *I* ^2^ = 0%	4.06 (0.18, 93.41) *I* ^2^ = 92%
Congenital malformations	6.66 (3.26, 13.61)^a^ *I* ^2^ = 6%	*z*‐score 1.72 *p*‐value 0.09^b^	5.22 (2.39, 11.39)^a^ *I* ^2^ = 40%	6.66 (3.26, 13.61)^a^ *I* ^2^ = 6%

Abbreviations: *I* ^2^, heterogeneity assumption; LGA, large for gestational age; N/A, not applicable; OR, odd ratio; PCOS, polycystic ovary syndrome; SGA, small for gestational age; SGA, small for gestational age.

^a^ Statistically significant.

^b^ Egger's test *p* < 0.10.

**FIGURE 8:**
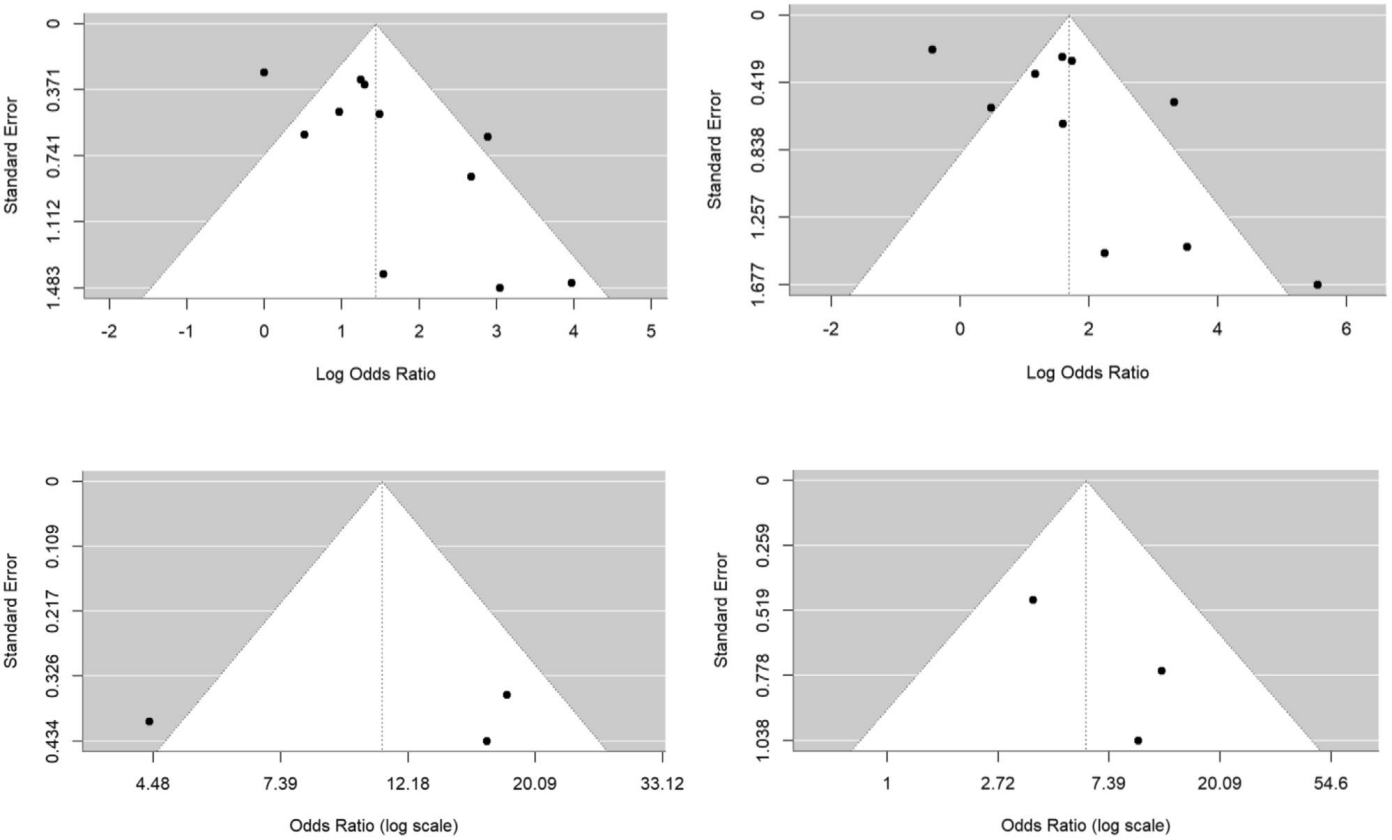
Funnel plots for the associations with possibility of publication bias. The funnel plots show the studies reporting associations between GDM and DVM (top left), DVM and stillbirth (top right), DVM and preterm birth (bottom left), and DVM and congenital malformations (bottom right).

Based on the Egger test, the associations of DVM with GDM, congenital malformations, stillbirth, and preterm birth had a risk of publication bias (*p* < 0.10; Figure 8).

Sensitivity analyses were performed for each association, limiting included studies to those with low NOS scores. The associations between DVM and GDM (OR = 4.90; 95% CI = 2.98, 8.06; *I* ^2^ = 39%), DVM and congenital malformations (OR = 5.22; 95% CI = 2.39, 11.39; *I* ^2^ = 40%), DVM and stillbirth (OR = 4.89; 95% CI = 3.55, 6.72; *I* ^2^ = 0%), and DVM and preterm birth (OR = 17.41; 95% CI = 10.14, 29.90; *I* ^2^ = 18%) all persisted with *I* ^2^ less than 50%.

In a sensitivity analysis focusing on studies in which the placental histopathology definition was according to the Amsterdam consensus criteria, the associations persisted only for stillbirth (OR = 12.06; 95% CI = 3.40, 42.78; *I* ^2^ = 50%) and congenital malformations (OR = 6.66; 95% CI = 3.26, 13.61; *I* ^2^ = 6%) (Table 5). No studies reporting maternal obesity used the Amsterdam criteria for placental histology assessment. All placentas reported in studies examining congenital malformations were assessed by Amsterdam criteria.

## 4 Discussion

### 4.1 Main Findings

In this meta‐analysis, maternal conditions associated with insulin resistance, including GDM, PGDM, and maternal obesity, as well as congenital malformations, were the main risk factors associated with DVM when broad criteria were used, including studies pre‐Amsterdam consensus.

With regard to adverse pregnancy outcomes, meta‐analysis of all studies showed an association with stillbirth as well as preterm birth when a broader definition of DVM was used.

In sensitivity analyses based on NOS low risk of bias and Egger's test, GDM, PGDM, maternal obesity, and foetal congenital malformations were associated with DVM, as well as stillbirth and preterm birth. When limiting meta‐analysis to DVM according to the Amsterdam consensus criteria, the associations persisted only for stillbirth and congenital malformations (Table 5).

### 4.2 Strengths and Limitations

This is the first comprehensive systematic review and meta‐analysis of studies that classify and report DVM‐related risk factors and outcomes. A potential limitation of this review is that we included studies with various definitions of DVM. Assessment of placental maturity by histology is still challenging for pathologists. Blinding is important [74], but information on gestational age at delivery is necessary for making the diagnosis. More robust and accurate definitions of DVM are important, as there are still variations and disagreements between pathologists. We conducted sensitivity analyses focusing on studies that explicitly used the Amsterdam criteria. Another limitation was that most of the studies included in the meta‐analysis did not consistently report adjusted odds ratios.

### 4.3 Interpretation

Our findings are consistent with a recent systematic review study of placental lesions found in diabetes, which found that increased villous immaturity and angiogenesis measures were associated with diabetes during pregnancy [75]. Impaired insulin function and glucose dysmetabolism mechanistically affect placental villous‐vascular tree development in affected pregnant women. Circulating angiogenic factors, fibroblast growth factors, peroxisome proliferator‐activated receptors, and placental growth factors are known to regulate placental development and normal villous maturation [76, 77]. In placentas with DVM, the findings related to angiogenic factors are inconsistent [39, 78, 79].

Some other less highlighted maternal risk factors, such as hypertensive disorders of pregnancy, polycystic ovary syndrome (PCOS), and opioid maintenance therapy, were also reported with increased likelihood of placental DVM in a few studies [21, 24, 34, 35, 43, 45]. However, studies reporting DVM in association with hypertension and opioid maintenance therapy were not blinded. In pregnancies with hypertension and spiral artery remodelling defects, the placental dysfunction tends to be accelerated rather than delayed villous maturation [80, 81]. Burke implied that parity is associated with distinct villous maturation patterns [24], with a higher prevalence of accelerated villous maturation and DVM in nulliparous and parous women, respectively. However, parity is associated with hypertension and diabetes in turn. In Koster's study, PCOS was a risk factor for villous immaturity, thrombosis, and the presence of nucleated red blood cells in the placenta [35]. Women with PCOS are, however, more likely to exhibit obesity and insulin resistance, predisposing them to GDM [82, 83, 84]. DVM has also been associated with possible undiagnosed glucose dysmetabolism, not fulfilling the diagnostic criteria for GDM by nature of a lower severity or atypia [14, 22, 85]. Pregnancies with LGA have also been associated with villous immaturity [63]. The common risk factor may be glucose dysmetabolism.

Our meta‐analysis has found that congenital foetal malformations are associated with DVM, including when limited to studies using Amsterdam consensus criteria. The link between congenital malformations and maternal diabetes is well known [86, 87]. A meta‐analysis demonstrated that the risk of major congenital malformations is even higher in GDM pregnancy than in PGDM and non‐diabetic pregnancies [88]. A possible explanation for this association is that undiagnosed and untreated pre‐existing diabetes may cause both malformations and DVM, but this is hypothetical. Because the number of studies reporting placental villous maturation in congenital malformations was small, we did not perform a subgroup analysis by malformation type.

Foetal macrosomia has also been reported in association with placentas with larger villous diameter, stroma, and membrane thickness that histologically mimic DVM lesions [89] or are formally diagnosed as DVM [63]. Higgins et al. reported that babies born to women with diabetes and DVM had a higher neonatal weight than those without [32]. In contrast, Beaudet reported a significantly higher proportion of DVM in the placentas of small, vulnerable newborns than in those of normally grown counterparts, but only in the presence of other pathologies related to growth restriction, such as maternal vascular malperfusion [50]. In DVM, the placenta promotes angiogenesis to compensate for inefficient maternal‐foetal exchange, although reduced blood flow may still occur. An evaluation using 3D power Doppler ultrasound of the diabetic placenta found a reduction in the ‘blood flow index’ in the late third trimester [40]. Due to a possible longstanding hypoxic‐ischemia in placentas with DVM, there might be an increased level of foetal erythropoietin [2, 90]. As a consequence of post‐placental foetal hypoxemia, the placenta increases the formation of distal villi to maintain optimal foetal oxygenation [91, 92]. The extensive formation of distal villi may occur concomitantly with the upregulation of nutrient transporters in the syncytiotrophoblast to maintain foetal nutrient supply, despite a failure to deliver oxygen to the foetus. This possibly explains the finding of sudden and unexpected stillbirth following a routine growth scan in pregnancies with diabetes and DVM.

Regarding adverse outcomes associated with DVM, our meta‐analysis found that the histologic appearance of DVM was associated with poor outcomes, including stillbirth and preterm birth, which reflects the functional impairment of affected placentas. With regard to preterm birth, the diagnosis of DVM in preterm placentas is controversial. It may relate to overdiagnosis, particularly if pathologists are not blinded to outcome. Even with the Amsterdam criteria, the histopathological diagnosis of DVM is inconsistent, with poor interobserver agreement between pathologists [93]. In DVM, the distance between the intervillous space and foetal capillaries is increased, and a longer placental oxygen diffusion distance can predispose babies to suffer more profoundly from additional stressors [89, 94, 95] which likely explains why some, but not all, babies with DVM are stillborn. Helgadottir et al. used a Causes of Death and Associated Conditions classification system to stratify perinatal death causes and found that placenta histopathology is found in most stillbirth cases. In mothers with idiopathic or unexplained stillbirth, without any reported placental pathologies, smoking history and diabetes appear to be prevalent [96]. An investigation on 15 years of singleton stillbirths in Thailand reported DVM in 13% of placental‐related stillbirths, with DVM more limited to late third‐trimester stillbirths [97].

Different types and manifestations of maternal glucose dysmetabolism appear associated with DVM. Some authors have found that with early diagnosis and optimal glycaemic control, histomorphology changes were less prevalent in women with diabetes than in controls [98]. It is plausible and likely that good control will help prevent DVM. Even though DVM is a condition mostly associated with term pregnancy, the best window for intervention may start earlier, particularly if preterm birth and congenital malformations are to be reduced.

## 5 Conclusion

Maternal dysmetabolism, including GDM, PGDM, and maternal obesity, appears to be the leading risk factor associated with DVM, when broad criteria are used, which in turn is associated with significant harm to the baby, including potentially stillbirth and preterm birth. The association of DVM with congenital abnormalities poses a question as to whether maternal dysmetabolism predates the pregnancy, manifesting as PCOS, obesity, or other types of insulin resistance not explicitly diagnosed as diabetes. As the existing literature appears prone to risk of bias and clinical heterogeneity, a large prospective study with robust diagnosis of DVM is needed to explore the association between maternal dysmetabolism, congenital malformations, DVM, and pregnancy outcomes. Future research should also examine the management and outcomes of subsequent pregnancies.

## Author Contributions

Muhammad Pradhiki Mahindra, Sara L. Hillman, and Dimitrios Siassakos conceived the study and designed the review protocol. Muhammad Pradhiki Mahindra and Muhammad Pradhika Mapindra performed the literature search and screened the articles. Muhammad Pradhiki Mahindra and Muhammad Pradhika Mapindra performed the risk‐of‐bias assessment and data extraction from the included studies. Muhammad Pradhiki Mahindra, Muhammad Pradhika Mapindra, and Dimitrios Siassakos synthesised the data. Muhammad Pradhiki Mahindra wrote the first draft of the manuscript. Hadi Waheed, Owen Vaughan, Sara L. Hillman, and John Ciaran Hutchinson critically revised successive drafts of the manuscript. Dimitrios Siassakos and John Ciaran Hutchinson are the guarantors of the review.

## Funding

The authors have nothing to report.

## Conflicts of Interest

The authors declare no conflicts of interest.

## Supporting information


**Table S1:** Search strategy and article screening process.

## Data Availability

Data extracted from the included paper is available upon request to the corresponding author.
