# Supplementary material for: Risk Factors and Outcomes Associated With Delayed Villous Maturation in Placenta: A Systematic Review and Meta‐Analysis
Source: BJOG. 2026 Jan 12;133(5):900–21. doi: 10.1111/1471-0528.70125 (PMC12972855; doi:10.1111/1471-0528.70125)
Supplement: Supplementary file 1 — Table S1: Search strategy and article screening process. [file BJO-133-900-s001.docx]

**Table S1. Search strategy and article screening process**

| **Medline** | 590 |  |  |  |
| --- | --- | --- | --- | --- |
| **EMBASE** | 293 |  |  |  |
| **Web of Science** | 1386 |  |  |  |
| **MIDIRS** | 27 |  |  |  |
| **Total records identified** | **2296** |  |  |  |
| **Primary article screening** | **151** | **Included** | **2145** | **Excluded:**   - **Duplicate** (n=850) - **Review articles** (n=32) - **Animal study** (n=259) - ***In-vitro* study** (n=348) - **Case report/series** (n= 307) - **Birth cohort** (n= 129) - **RCT or clinical trial studies** (n= 220) |
| **Study Eligibility screening** | **78** | **Included** | **73** | **Excluded:**   - **Study design out of scope/ not appropriate for inclusion** (n=28) - **Target population out of scope/ not appropriate for inclusion** (20) - **Conference proceedings, abstract, poster** (n=22) - **Comments to journal editor** (n=3) |
| **Full-text assessment screening** | **52** | **Included** | **25** | **Excluded:**   - **Unclear definition of DVI/ DVM (**n=2) - **Single study group** (n=6) - **Studies not comparable on study design or analysis** (n=8) - **Placenta stereology or histologic morphometry studies** (n=9) |
| **Risk of bias assessment** | **52** | **Included** | **0** | **Excluded:** |

**Ovid MEDLINE(R)**

ALL <1946 to December 04, 2023> https://ovidsp.ovid.com/ovidweb.cgi?T=JS&NEWS=N&PAGE=main&SHAREDSEARCHID=3tt3zQO2QNalQMvu9L2ulJpMxB7yvVKezo1R3ZLp8pyOQbRcSrefKQHEtemjOYntg

1 Pathology/ 33175

2 immatur*.mp. 94785

3 villous.mp. or Placenta/ 72447

4 2 and 3 540

5 delayed villous maturation.mp. 40

6 distal villous immaturity.mp. 4

7 villous maturation defect.mp. 2

8 variable villous maturation.mp. 0

9 villous dysmaturity.mp. 10

10 4 or 5 or 6 or 7 or 8 or 9 588

11 (4 or 5 or 6 or 7 or 8 or 9) not animal'.mp. 552

12 maturitas retardata.mp. 0

13 terminal villous deficiency.mp. 0

14 peripheral villous immaturity.mp. 0

15 irregular villous maturation.mp. 0

16 disorder of villous development.mp. 1

17 defective villous maturation.mp. 2

18 10 or 11 or 12 or 13 or 14 or 15 or 16 or 17 **590**

**Embase**

<1974 to 2023 December 28>

https://ovidsp.ovid.com/ovidweb.cgi?T=JS&NEWS=N&PAGE=main&SHAREDSEARCHID=24HFgrwSSoHzKEtmtUhAvr20ghEnMPvnBfNA0QjdfKHU7oZUYDtahfMmktcK3zZMO

1 delayed villous maturation.mp. 83

2 distal villous immaturity.mp. 13

3 villous maturation defect.mp. 2

4 variable villous maturation.mp. 0

5 villous dysmaturity.mp. 15

6 exp immaturity/ or immatur*.mp. 117337

7 villous.mp. 19981

8 placenta.mp. or placenta/ 136788

9 7 and 8 5312

10 6 and 9 198

11 1 or 2 or 3 or 4 or 5 or 10 289

12 maturitas retardata.mp. 1

13 terminal villous deficiency.mp. 0

14 peripheral villous immaturity.mp. 0

15 irregular villous maturation.mp. 0

16 disorder of villous development.mp. 2

17 defective villous maturation.mp. 2

18 11 or 12 or 13 or 14 or 15 or 16 or 17 **293**

**Maternity & Infant Care Database (MIDIRS)**

<1971 to December 12, 2023>

https://ovidsp.ovid.com/ovidweb.cgi?T=JS&NEWS=N&PAGE=main&SHAREDSEARCHID=1CA5YwjGDIqhyxCBBVG7hc3jdcreZNNJgexx9HcCkpJhuQ1BmRgDj9GoeY31phxOp

1 delayed villous maturation.mp. 9

2 distal villous immaturity.mp. 0

3 villous maturation defect.mp. 0

4 variable villous maturation.mp. 0

5 villous dysmaturity.mp. 2

6 placenta.mp. [mp=abstract, heading word, title] 7790

7 (villous or villi).mp. [mp=abstract, heading word, title] 1194

8 (immatur* or dysmatur*).mp. [mp=abstract, heading word, title] 1523

9 6 and 7 390

10 8 and 9 14

11 1 or 2 or 3 or 4 or 5 or 10 25

12 maturitas retardata.mp. [mp=abstract, heading word, title] 0

13 terminal villous deficiency.mp. [mp=abstract, heading word, title] 0

14 peripheral villous immaturity.mp. [mp=abstract, heading word, title] 0

15 irregular villous maturation.mp. [mp=abstract, heading word, title] 0

16 disorder of villous development.mp. [mp=abstract, heading word, title] 1

17 defective villous maturation.mp. [mp=abstract, heading word, title] 1

18 11 or 12 or 13 or 14 or 15 or 16 or 17 **27**

**Web of Science**

((((((((((((((((ALL=(defective villous maturation)) OR ALL=(disorder of villous development)) OR ALL=(irregular villous maturation)) OR ALL=(peripheral villous immaturity)) OR ALL=(maturitas retardata)) OR ALL=(terminal villous deficiency)) OR ALL=(delayed villous maturation)) OR ALL=(distal villous matur*)) OR ALL=(placental maturation defect*)) OR ALL=(villous dysmatur*)) OR ALL=(placental matur*)) OR ALL=(variable villous maturation)) OR ALL=(villous immatur*)) OR ALL=(immature villous)) OR ALL=(placental maturation defect*)) OR ALL=(villous dysmatur*)) AND ALL=(placenta) **1386**
